# NLRP3 inflammasome-mediated cytokine production and pyroptosis cell death in breast cancer

**DOI:** 10.1186/s12929-021-00724-8

**Published:** 2021-04-12

**Authors:** Sara Socorro Faria, Susan Costantini, Vladmir Cláudio Cordeiro de Lima, Victor Pianna de Andrade, Mickaël Rialland, Rebe Cedric, Alfredo Budillon, Kelly Grace Magalhães

**Affiliations:** 1 Laboratory of Immunology and Inflammation, Department of Cell Biology, University of Brasilia, Brasilia, DF Brazil; 2 Experimental Pharmacology Unit - Laboratory of Mercogliano (AV), Istituto Nazionale Tumori-IRCCS Fondazione G. Pascale, 80131 Naples, Italy; 3 Department of Medical Oncology and Laboratory of Translational Immuno-Oncology, A.C. Camargo Cancer Center, São Paulo, Brazil; 4 Department of Pathology, A.C. Camargo Cancer Center, São Paulo, Brazil; 5 Platform of Transfer in Cancer Biology, Centre Georges François Leclerc, 21000 Dijon, France; 6 Institut National de la Santé et de la Recherche Médicale (INSERM) UMR 1231, 21000 Dijon, France; 7 UFR Sciences de la Vie, Terre et Environnement, Université de Bourgogne Franche-Comté, 21000 Dijon, France

**Keywords:** Breast cancer, Gasdermins, IL-1β, NLRP3 inflammasome

## Abstract

Breast cancer is the most diagnosed malignancy in women. Increasing evidence has highlighted the importance of chronic inflammation at the local and/or systemic level in breast cancer pathobiology, influencing its progression, metastatic potential and therapeutic outcome by altering the tumor immune microenvironment. These processes are mediated by a variety of cytokines, chemokines and growth factors that exert their biological functions either locally or distantly. Inflammasomes are protein signaling complexes that form in response to damage- and pathogen-associated molecular patterns (DAMPS and PAMPS), triggering the release of pro-inflammatory cytokines. The dysregulation of inflammasome activation can lead to the development of inflammatory diseases, neurodegeneration, and cancer. A crucial signaling pathway leading to acute and chronic inflammation occurs through the activation of NLRP3 inflammasome followed by caspase 1-dependent release of IL-1β and IL-18 pro-inflammatory cytokines, as well as, by gasdermin D-mediated pyroptotic cell death. In this review we focus on the role of NLRP3 inflammasome and its components in breast cancer signaling, highlighting that a more detailed understanding of the clinical relevance of these pathways could significantly contribute to the development of novel therapeutic strategies for breast cancer.

## Background

Breast cancer is the most common neoplasia in women worldwide and is the second cause of cancer death [1]. This cancer is very heterogeneous having different morphological, phenotypic and molecular characteristics [2]. Its molecular subclassification is primarily based on the expression of the estrogen receptor (ER), the progesterone receptor (PR) and the human epidermal growth factor (HER2) [3]. In fact, the tumors are classified as hormone-positive (ER+ , PR+) or HER2-positive (HER2+) when they have expression of ER and/or PR, or over-expression HER2, respectively [3]. The tumors ER/PR and HER2 negative are classified as triple-negative breast cancer (TNBC), having high genomic instability and mutational burden what, potentially generates neoantigens [4] and attracts tumor-infiltrating lymphocytes (TILs) [5].

According to the PAM50 classification, five distinct molecular subtypes of breast cancer (Luminal A, Luminal B, HER2-enriched, basal-like and normal-like) [3] were established and correlate with prognosis and can guide treatment decisions and the enrollment of patients in clinical trials. As a proxy to the PAM50 classification, usually an immuno-histochemistry based classification is more used in clinical practice. In detail, luminal A is HER2^−^, ER^+^ and/or PR^+^, with low levels of Ki-67; luminal B is ER^+^ and/or PR^+^ (or PR low < 20%), HER2^−^ or HER2^+^, with high expression of Ki-67; triple-negative (the majority of triple-negative tumors are basal-like) is HER2^−^, ER^−^ and PR^−^;HER2-enriched is hormone-receptor negative (estrogen-receptor and progesterone-receptor negative) and HER2 positive; normal-like is similar to luminal A (HER2, ER^+^ and/or PR^+^, with low levels of Ki-67) with a different expression pattern and worse patient outcome. Moreover, in 2012 an accurate genomic analysis based on a combination of gene expression profiles and copy number aberrations led to a further subclassification of breast cancers into 10 clusters, although this has not been incorporated into clinical practice yet [6].

Historically, breast cancer was defined as immunologically “cold” and quiescent because it has low tumor lymphocyte infiltration, low tumor mutational burden, and low anti-PD-1/L1 response in comparison to other cancers [7]. More recently, plenty evidence demonstrated that the breast cancer immune landscape is very heterogeneous and dynamic, with significant variation observed across patients, subtypes and disease settings. This highlighted the importance of tumor microenvironment elements (composed of cytokines, diverse immune cells and stroma) in modulating the immune response against breast cancer [8].

In general, the immune system is divided into two different responses: innate immunity and adaptive immunity. The innate immune system plays a crucial role in perpetuating tumor cells, but also in triggering antitumor adaptive immune responses [9, 10]. Adaptive immunity is antigen-specific and develops immunological memory [11]. Besides, induction of non-specific memory in macrophages and monocytes exposed to damage-associated molecular patterns (DAMPs) or pathogen-associated molecular patterns (PAMPs) also develops and is called "trained immunity" [12].

In response to excessive inflammation, the innate immune system is activated by PAMPS or DAMPs, microbe-associated molecular patterns (MAMPs), and homeostasis-altering molecular processes (HAMPs) [13], which are recognized by pattern recognition receptors (PRRs) [14]. The classes of PRRs include Toll-like receptors (TLRs), nucleotide-binding oligomerization domain (NOD)-, leucine-rich repeat–containing receptors (NLRs), RIG-I-like receptors (RLRs), C-type lectin receptors (CLRs) and AIM-2 (absent in melanoma 2) like receptors [15, 16]. Although these groups differ in their structure, they converge on to similar signal transduction cascades, which trigger the activation of type I interferon and NF-κB [17]. In particular, it is important to underline that TLRs are the most defined PRRs and their interaction with different PAMPs induces intracellular signal transduction and activates innate immune associated genes, including inflammatory cytokines, costimulatory molecules and adhesion molecules [18]. Thus, TLRs act as participants in inflammation-induced tissue damage, leading to tumor progression or regression [19]. In this context, several data suggested a role of NLRP3 activation in breast cancer development [20, 21].

Considering that the mechanisms of inflammasome activation in tumor growth are specific to the type of cell, tumor tissue as well as the nature of their activators and inhibitors [22], in this review, we briefly summarize: (i) the critical role of inflammation in breast cancer microenvironment; (ii) the details related to the activation and regulation of the inflammasome; (iii) the description of NLRP3 and its components in breast cancer microenvironment; (iv) the involvement of immunogenic cell death in breast cancer; (v) the role of gasdermins and pyroptosis in breast cancer; and, finally, (vi) the therapeutic use of NLRP3 inflammasome components**.**

### Inflammation in breast tumoral microenvironment

Inflammatory signaling operates in many types of cancers, contributing to the induction of epithelial-to-mesenchymal transition, influencing epigenetic regulation, cellular plasticity, the generation of cancer stem cells (CSCs), and intra-tumoral heterogeneity [23–26]. The tumor microenvironment (TME) changes continuously over the course of tumorigenesis, due to mutations in malignant cells, the diverse nature of the microenvironmental composition and the different stromal cell proportions and their respective states of activation [27].

Inflammation can increase the risk of cancer promoting cells by infiltrating the TME, by releasing cytokines, growth factors, chemokines, and proangiogenic factors, and by generating genome instability and immune evasion [28]. It is well established that the systemic and local environment plays a tumor-initiating role through the generation of a persistent inflammatory response [29]. For example, obesity is associated with an increased risk for breast cancer development [30] and is characterized by a low-intensity systemic inflammatory response, as well, locally, by the appearance of *crown-like structures* (CLS) that consist of macrophage and phagocyted adipocytes, whose increased numbers strongly correlate with worse breast cancer prognosis [31].

Macrophages and fibroblasts are the most abundant cells in the breast tumor microenvironment [32]. Inflammatory responses are often accompanied by recruitment of fibroblasts and mesenchymal stem cells (MSCs) [33]. Notably, cancer-associated-fibroblasts (CAFs), the major stromal cells that contribute to the TME [34] in breast cancer, were analyzed in this disease at the single cell level and then categorized in different subclasses with different functional programs, and prognostic value [35]. It was also showed that disseminated breast cancer cells evoke phenotypic changes in lung fibroblasts, forming a metastatic niche, and that the disruption of the intercellular JNK-IL-1-CXCL signaling, reduced metastatic colonization, confirming an essential role of the crosstalk between breast cancer cells and their fibroblast niche in the progression of metastasis [36].

In breast cancer, macrophages represent up to 50% of the tumoral mass, becoming the main immune population [37]. Macrophage migration into tissues is controlled by many chemo-attractants. Among these, CCL2 (referred to as monocyte chemo-attractant protein-1, MCP-1) is the most important in tumor progression [38]. Tumor associated macrophages (TAMs) secrete cytokines, chemokines and enzymes that stimulate cell proliferation, tumoral progression and angiogenesis [39]. Besides, macrophages play a role in both innate and adaptive immunity by interacting with immune and epithelial cells to regulate the cellular environment through secretion of cytokines and chemokines [40, 41]. In the TME, cytokines are produced by a variety of cell types and exert their actions locally (autocrine and paracrine) or systemically by directly interacting with their specific membrane receptors [42].

The interleukin (IL)-1 family plays multifaceted roles in tumoral immunity [43, 44]. It includes seven ligands with pro-inflammatory activity (IL-1α, IL-1β, IL-18, IL-33, IL-36α, IL-36β, IL-36γ), as well as anti-inflammatory cytokines (IL-37 and IL-38) [43, 45], having crucial roles in host-defense responses, but also in inflammatory responses that contribute to cancer development [46]. In detail, IL-1β, IL-1α, and IL-18 are initially produced as precursors (pro-IL-1β, pro-IL-1α, and pro-IL-18). IL-1α and IL-1β bind to the same receptor (IL-1R) and recruit the IL-1R accessory protein [47, 48]. This process results in the activation of a cascade of immune and inflammatory genes [49].

Wallenstein et al. through an analysis of different genetically mouse models of breast cancer, evidenced that the loss of p53 in cancer cells induced the secretion of WNT ligands and stimulated TAMs to produce IL-1β, thus promoting a condition of systemic inflammation. Pharmacological inhibition of WNT secretion in p53-null breast cancer cells blocked macrophage-mediated IL-1β release, neutrophilic inflammation, and reduced metastasis formation [50]. Eyre et al., on the other hand, evidenced that IL-1β is produced by bone marrow cells and stimulates breast cancer colonization through autocrine WNT signaling [51].

**Fig. 1:**
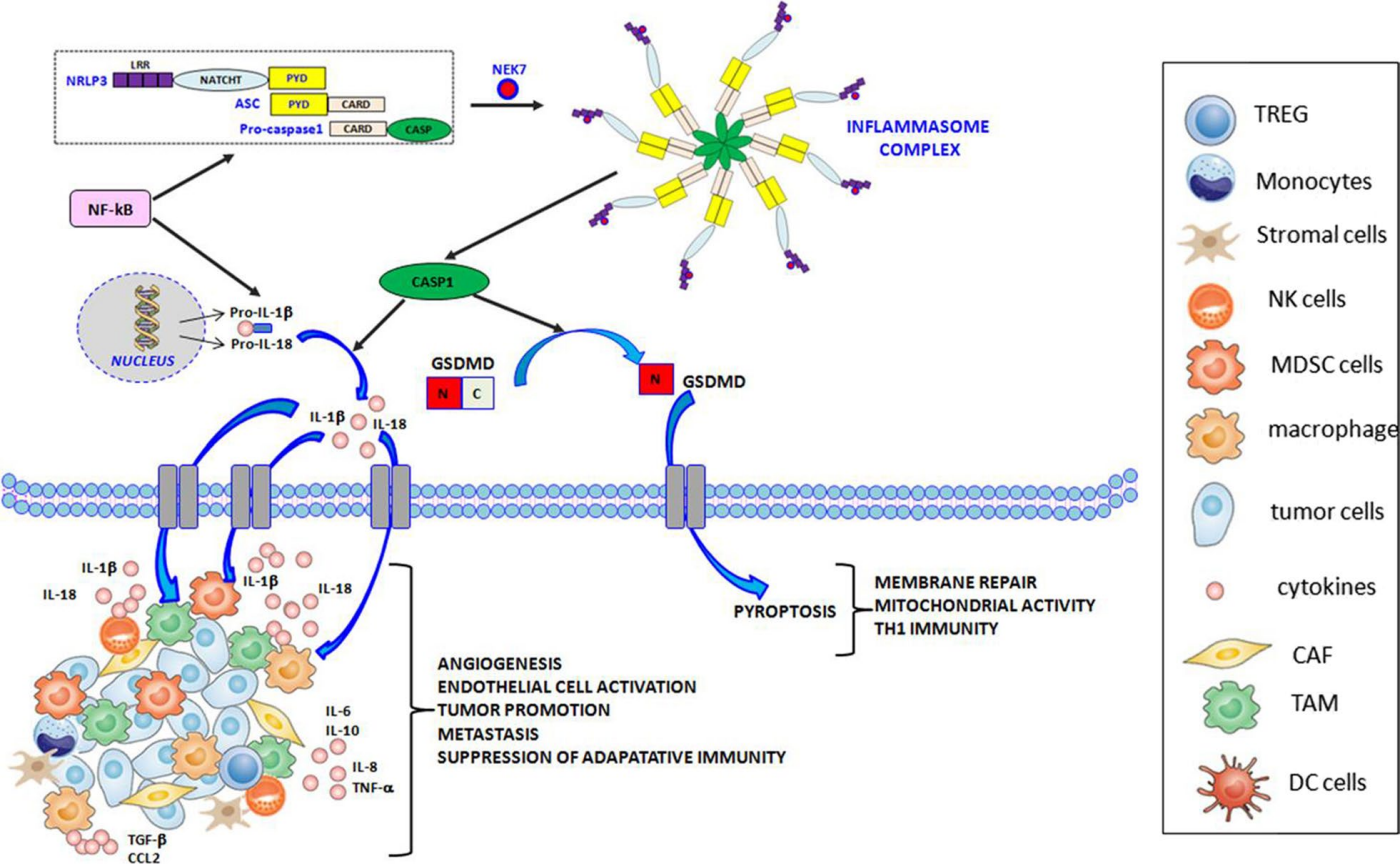
Inflammasome components and functions. After sensing specific stimuli, for example through NEK7, a member of the family of mammalian NIMA-related kinases (NEK proteins),the sensor NLR family pyrin domain containing 3 (NLRP3) assembles together with the adaptor apoptosis-associated speck-like protein (ASC) and the effector pro-caspase-1, via homotypic interactions between the N-terminal pyrin domain (PYD) domain of NLRP3 and the PYD domain of ASC, as well as between the respective Caspase Recruitment Domains (CARD) of ASC and pro-caspase-1. Assembly of the NLRP3 inflammasome leads to activation of caspase-1 (CASP1), which then cleaves the pro-forms of interleukin-1beta (IL-1β) and -18(IL-18), resulting in the secretion of biologically active cytokines, as well as gasdermin D (GSDMD), resulting in pyroptosis via the formation of pores at the plasma membrane. Inflammasomes are activated through different mechanisms and release IL-1β and IL-18 to initiate inflammation. In the figure we show all the cells like regulatory T cells (TREG), monocytes, stromal cells, natural killer (NK) cells, myeloid-derived suppressor cells (MDSC), macrophages, tumor cells, cancer-associated fibroblasts (CAF), tumor-associated macrophages (TAMS) and dendritic cells (DC) cells, able to release cytokines and chemokines such as IL-6 (interleukin 6), IL-8 (interleukin 8), IL-10 (interleukin 10), transforming growth factor-b (TGF-b), tumor necrosis factor-a (TNF-a) and C–C motif chemokine ligand 2 (CCL2). In details, cancerous cells and stromal cells can release chemokines and lead to neutrophil infiltration. Neutrophils will in turn secret more pro-inflammatory cytokines including interleukins and interferons. B cells and antibodies are also observable. TREG, TAMs, and MDSCs work together to enhance immunosuppression. The alteration of proinflammatory cytokines will lead to abnormal polarization of T helper cells

Both pro- IL-1β and pro-IL-18 are cleaved by the caspase-1, which influences Ca^2+^ and calpain-dependent processing of pro-IL-1α [52]. IL-1α, IL-1β, and IL-18 have been investigated in many types of cancer with both pro- and anti-tumorigenic functions [53] mediated by different cells (Fig. 1).

### Activation and regulation of inflammasome

The NLRP3 inflammasome is a multiple-protein complex comprising NLRP3, apoptosis-associated speck-like protein containing a caspase recruitment domain (ASC), and caspase 1. The activation of this complex, in turn, activates caspase 1, which cleaves pro-IL-1β and pro-IL-18, generating the mature forms of these inflammatory cytokines, IL-1β and IL-18 [54].

**Fig. 2:**
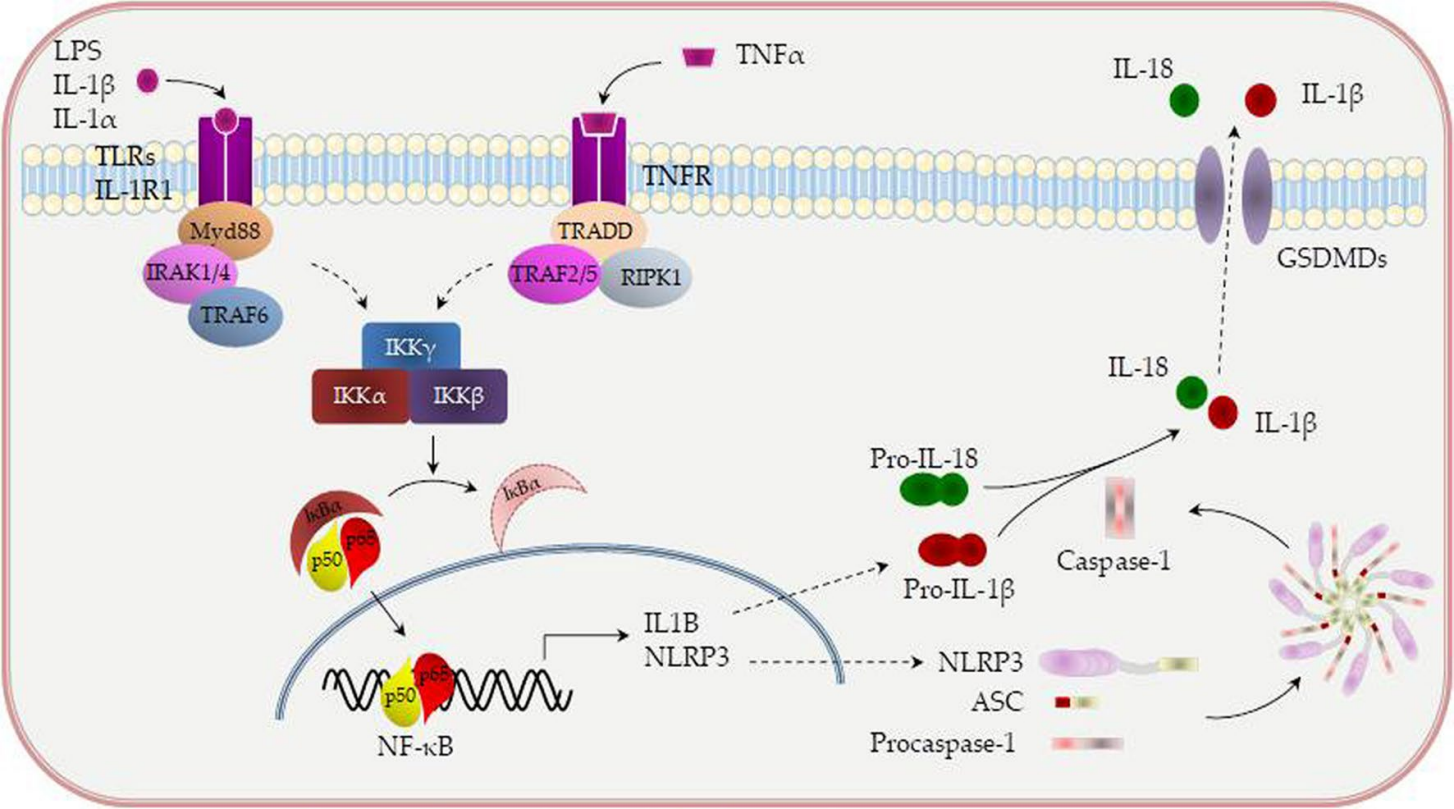
Canonical and non-canonical activation of NLRP3. NLRP3 (NOD-, LRR- and pyrin domain-containing 3) needs additional cofactors for the processing of interleukin-1β (IL-1β). MyD88/IRAK1/IRAK4 or TRIF activates TRAF6, which, in turn, catalyze the formation of a K63-linked polyubiquitin chain on TRAF6, itself. The polyubiquitin chain acts as a scaffold, recruiting TAK1 and its binding proteins, which, in turn, leads to IKK-α/β activation. Activated IKKα/β specifically phosphorylates IkBα, resulting in IkBα degradation and NF-kB translocation into the nucleus. TRIF can also recruit TRAF3 to activate TBK1 and IKKi. TBK1/IKKi directly phosphorylates IRF3/7 to activate type IFN I signaling pathway. Various molecules positively (green arrow) or negatively (red blunt arrow) regulate TLR-induced signaling pathways. TAK1 (transforming growth factor 1 activating kinase) restrains both NLRP3 priming and activation. TAK1 activity restricts NLRP3 priming by limiting spontaneous activation of receptor protein kinase 1 (RIP1). MyD88- myeloid differentiation primary response 88; IRAK1—Interleukin-1 receptor-associated kinase 1; IRAK4- Interleukin-1 receptor-associated kinase 4; TRIF- TIR-domain-containing adapter-inducing interferon-β; TRAF6- TNF receptor associated factor 6; TRAF3- TNF receptor associated factor 3; TBK1-TANK binding kinase 1

Two different types of NLRP3 activation have been described—canonical and non-canonical (Fig. 2). Canonical NLRP3 inflammasome activation demands two independent and parallel steps—transcription (priming) and oligomerization (activation) [55]. In the first phase, innate immune signaling via cytokine receptors, such as the tumor necrosis factor (TNF) receptor and/or TLR-adaptor myeloid differentiation primary response 88 (MyD88), promotes NLRP3 and pro-IL-1β transcription through the nuclear factor-kB (NF-kB) activation. In the second phase, the oligomerization of the NLRP3 with an apoptosis-associated speck-like protein containing a CARD (ASC) leads to pro-caspase-1 activation and IL-18 and IL-1β release [55, 56].

Different stimuli, including the inhibition of mitochondrial or glycolytic metabolism, viral RNA, post-translational NLRP3 modifications (such as ubiquitination and phosphorylation) and the degradation of extracellular matrix constituents may produce inflammasome activation and oligomerization. Moreover, the increase in potassium efflux through the cell membranes (i.e., exposure to pore-forming GSDMD, mixed lineage kinase domain-like protein activation, P2X7 purinergic receptor activation, cathepsin release and lysosomal damage), the up-regulation of mitochondrial reactive oxygen species (ROS), the following release of oxidized DNA from mitochondria and the cardiolipin externalization may induce NLRP3 inflammasome activation [56, 57].

Recently, a different non-canonical NLRP3 activation pathway, dependent on caspase-8, has been characterized [58, 59]. DAMPs and/or PAMPs may stimulate TLR4, leading to caspase-8 activation and its receptor. Receptor-interacting protein 1 (RIP1)–fatty acid synthase and (FAS)-associated death domain (FADD) protein may induce both canonical NLRP3 activation and the transcription step.

Analysis of the signaling pathways downstream of priming by TLRs has demonstrated that the adapter molecule MyD88 and the IL-1 receptor-associated kinases IRAK-1 and IRAK-4 are crucial for fast NLRP3 priming [60, 61]. Liposaccharide (LPS) sensed by TLR4 activates IRAK1 and IRAK4 via MyD88 and so priming of NLRP3 is consistent with known TLR responses [62]. Pharmacological inhibitors of NF-κB and of protein synthesis fail to block rapid NLRP3 activation [63, 64] indicating that posttranslational modifications (PTMs) are regulators of NLRP3 activation.

As the mechanism of NLRP3 inflammasome assembly is not understood, it is uncertain whether these PTMs may affect specific steps in this process, such as NLRP3 oligomerization or structural changes that may be required for NLRP3 activation [65].

Activation of the innate immune response through TLR- and NLR- signaling pathways serves as a link between chronic inflammation and cancer [66, 67]. Chronic inflammation, caused by abnormal NF-κB or inflammasome activation, is associated with cancer through PRRs-mediated cytokine production [68, 69]. Recent studies demonstrate that PRRs and their regulators have both favorable and unfavorable effects on cancer cells [70]. On one hand, some PRRs induce an anti-tumor immune response to inhibit tumor progression [71]. However, uncontrolled innate immune signaling may provide a microenvironment for cancer cell proliferation and immune surveillance evasion.

Several reports have shown that, when present, transforming growth factor 1 activating kinase (TAK1) is considered an important component of proinflammatory signaling through activation of NF-κB downstream of TNFR1 or TLR receptor activation, where it acts downstream of ubiquitinated RIP1 or TRAF6, respectively [72]. Given its role in these inflammatory pathways, TAK1 inhibitors can act as attractive targets for therapy against cancer, due TAK1 being crucial to many cell death pathways. The involvement of NLRP3-driven inflammation adds extra impetus to such treatment as it has been shown that NLRP3 activation in various tissues can activate NK cell responses, which could contribute to the clearance of tumor [73]. Thus, the dual function of a TAK1 inhibitors to cause cancer cell death while simultaneously activating NLRP3 makes them a potential powerful anticancer agent for breast cancer [74].

### NLRP3 and its components in breast cancer microenvironment

Inflammation and immunosuppression are associated synergistically promoting tumor development [30]. In the context of breast cancer, it has been demonstrated that IL-1β and NLRP3 were overexpressed in the breast tumor microenvironment concomitantly to the accumulation of MDSCs and TAMs [75]. Moreover, the analysis of The Cancer Genome Atlas (TCGA) dataset showed that *CASP1* expression was increased in basal and luminal B type tumors of patients with obesity, whereas there is no correlation between obesity and *CASP1* expression in HER2 or Luminal A subtypes breast cancer [76]. In silico meta-analysis of the expression of the genes *CASP1*, *NLRP3* and *IL-1β* in breast cancer exhibited an association with prolonged overall survival [77]. Similarly, *NLRP3* had a positive correlation with survival in all molecular subtypes from the TCGA breast cancer dataset [76]. On the other hand, NLRP3 and IL-1β expression in TAMs correlated with survival, lymph node invasion, and metastasis in patients with HER2^+^ breast cancer [78].

Kersten et al. evidenced, through gene expression analysis, that the expression of *CCL2* and *IL1Β* transcripts was highly enriched in basal-like tumors when compared to other subtypes of human breast cancer [79]. In a mouse model of basal-like breast cancer, a complex Notch-dependent paracrine loop between tumor cells and TAMs promoted an immunosuppressive tumor microenvironment, regulating the expression of IL-1β and CCL2 [80]. It has been shown both pro-metastatic and anti-metastatic roles for IL-1 signaling in models of breast cancer [81].

The divergent IL-1 responses in metastasis may be explained by different signal interactions in the different breast cancer subtypes [82]. For instance, in basal-like, transcriptional reprogramming of tumor-infiltrating myeloid cells was mediated in part by IL-1β induced NF-kB activation, indicating that this subtype can adapt to and take advantage of inflammatory signaling to grow and metastasize [83]. These observations suggest that interrupting this paracrine interaction through inhibition of CCL2 and IL-1β could have therapeutic applications in basal-like TNBC.

In a recent study, it was found that fibroblasts sense DAMPs and, in response, activates the NLRP3 pathway, resulting in a pro-inflammatory signaling and secretion of IL-1β in mouse and human breast carcinomas. Functionally, cancer associated fibroblast-derived inflammasome promoted tumor progression and metastasis by modulating the TME in an immunosuppressive manner by regulating the expression of adhesion molecules and stimulating endothelial cells. These findings elucidate the mechanism by which CAFs can promote breast cancer progression and metastasis through the physiological tissue damage response of fibroblasts [84].

In 2020, the precise regulatory mechanisms that dictate IL-1β over-expression were investigated. It was demonstrated that the co-culture of human monocyte-like cells and TNBC cells increases IL-1β secretion, and that TNBC conditioned-media induces IL-1β production in macrophages. Besides, macrophage depletion proved to be able to decrease serum IL-1β, and to block breast cancer progression in an orthotopic breast cancer mouse model [85].

Interestingly it has also been demonstrated that, in murine invasive breast cancer models, the absence of a functional NLRP3 impaired tumor growth, and NK cell depletion abolished the anti-tumoral effect independently from effector mechanisms of IL-1β and IL-18 [86].

### Immunogenic cell death in breast cancer

Immunogenic cell death (ICD) results from exposure to chemical, physical or infective agents [85], which promotes both intracellular stress, mediated by reactive oxygen species (ROS), and structural alterations to the endoplasmic reticulum (ER), leading to release of DAMPs and cytokines, which, in turn, alter the TME and the influx of tumor infiltrating lymphocytes (TILs) [87]. Although apoptosis appears to represent the predominant mechanism of ICD, other modes of ICD have also been described, including necroptosis, ferroptosis and pyroptosis [19].

A substantial number of studies have shed light on the dynamic molecular and cellular mechanisms that underlie the ability of cancer cells to undergo ICD. Ladoire et al. investigated the expression of nuclear HMGB1 and the occurrence of cytoplasmic LC3B (microtubule-associated protein 1 light chain 3B)-positive puncta, which is an immunohistochemical sign of autophagy, in tumor tissues from breast cancer patients who were treated with adjuvant chemotherapy. The results showed that the combination of nuclear HMGB1 and positivity for LC3B(+) puncta was an independent prognostic marker significantly associated with metastasis-free survival and improved breast cancer-specific survival [88]. Further investigation demonstrated that the loss of HMGB1 and the blockade of autophagy have a negative impact on anticancer immunosurveillance.

The antitumor effects of inflammatory cell death demonstrated, for example, that induction of pyroptosis or necroptosis by inflammasome agonists or DAMP-mediated activation of RIPK3 enhances immune cell responses and reduces tumor growth [89]. In certain contexts, inflammatory cell death may also be pro-tumorigenic. This is consistent with the complex role of inflammation in both enhancing and inhibiting tumor growth, and indicated that the elements, timing and levels of induction need to be monitored.

### Gasdermins and pyroptosis in breast cancer

As reported above, caspase-1, after being activated by the NLRP3 inflammasome, is capable to proteolytically cleaving GSDMD. In humans, gasdermins (GSDMs) consist of the following six members: GSDMA, GSDMB, GSDMC, GSDMD, GSDME (also known as deafness autosomal dominant 5-DFNA5) and DFNB59, having pyroptosis-inducing activity [90].

It is important to highlight that pyroptotic death is an inflammatory form of programmed cell death characterized by cellular swelling and rupture, lysis, nuclear condensation, DNA fragmentation and IL-1β and IL-18 leakage [91], exacerbating the inflammatory response in the extracellular space [92]. Pyroptosis induces DAMPs, such as HMGB1, IL-1α, and adenosine-triphosphate (ATP) [93, 94], release and, hence, promotes a local immune response [95]. These molecules are involved in many types of cancer and contribute to the tumorigenic potential of inflammasome activation [96]. On the other hand, pyroptosis-induced products can also limit the survival of tumor cells, and trigger, through immunogenic signals, the activation of the innate immune response blunting cancer progression [22].

GSDMD is expressed in different human tissues and subsets of leukocytes [97]. Expression of GSDMD is transcriptionally regulated by IFN regulatory transcription factor-2 (IRF2) [98]. IRF2 regulation of GSDMD levels may represent a critical regulatory axis in cancer cell death, given that its loss in cancer leads to immune evasion and resistance to immunotherapy [99]. GSDMD and GSDME are cleaved into N-terminal and C-terminal fragments by inflammatory caspases such as caspase-1, -4, -5, -11, and caspases-3 and -8, in the case of GSDME [100, 101]. When TAK1 or IKK signaling is inhibited, caspase-8 can also cleave GSDMD to drive pyroptotic cell death [89].

Some authors evidenced that the retinoic acid-inducible gene (RIG-I) signaling is capable of inducing pyroptosis through of the activation of the intrinsic apoptosis pathway, of caspase-1 and of GSDMD in ER^+^ breast cancer cells [102].

Gasdermin E (GSDME) is cleaved by caspase-3 and its necrotic fragment is able to regulate apoptotic cell disassembly and progression to secondary necrosis, providing a molecular mechanism for secondary necrosis. Through this mechanism, GSDME forms pores and leads to the release of DAMPs and cytokines [103], amplifying the cell death signal. Zhang et al. identified that 91% (20 of 22) of the mutations in the gene encoding *GSDME* from cancer patients result in loss of function. Tumor cells expressing wild-type *Gsdme* were markedly impaired in their ability to proliferate compared to cells expressing the mutant form of *Gsdme*, highlighting the importance of pore-forming activity of GSDME in the control of tumor growth [104].

*GSDME* also has been identified as a possible tumor suppressor gene [105]. Moreover, its methylation was proposed as a biomarker for breast cancer detection and its increased methylation in histologically normal breast tissue surrounding the tumor was suggested as a good early detection marker [106]. In another study conducted by the same authors, *GSDME* methylation resulted in lower expression of its protein in breast cancer cells compared to normal cells, and in the inability to activate pyroptosis in tumor cells [107]. Furthermore, its silencing led to greater migratory capacity of tumor cells [108], suggesting that the regulation of this gene is complex and not completely elucidated yet.

In a recent study, aberrant *GSDME* methylation was seen pan-cancer, with widespread hypermethylation in promoter CpGs and hypomethylation of gene body CpGs. These aberrant methylation patterns were used in a model that could segregate cancer from normal samples and predict the tumor type with accuracy [109]. GSDME is highly expressed in normal cells and is low in tumor cells due to the hypermethylation of *GSDME* promoter. When the expression of GSDME was low in tumor cells, the DNA methyltransferase inhibitor decitabine could inhibit the hypermethylation of its gene promoter, thus leading to pyroptosis of tumor cells [110].

Using TCGA database, Zhang et al. identified single-nucleotide polymorphisms of *GSDME* and tested triple-negative breast and colorectal cancer cell lines expressing the mutant proteins for their ability to undergo pyroptosis. Tumors that were *GSDME*^*–/–*^ had fewer TILs and NK cells. The authors also observed that granzyme B cleavage of GSDME resulted in increased antitumor immunity and reduced tumor growth [104]. Preclinical evidence suggests that enhanced immune infiltration through pyroptosis augmented response to therapy [111]. These findings are of particular interest given that immunotherapeutic strategies often seek to improve T-cell responses against cancer cells.

Moreover, other studies showed that *GSDMD/GSDME* mRNA methylation induces decreased GSDMD/GSDME expression levels in tumor cells when compared with normal cells, making it difficult to activate pyroptosis in tumor cells [112]. Thus, GSDMD and GSDME may alter the local TME via proinflammatory pathways.

Hou et al. reported that nuclear PD-L1 induces pyroptosis in breast cancer cells through gasdermin C (GSDMC) under hypoxia conditions; critically, they found that this leads to tumor necrosis, affecting the prognosis of patients. Additionally, the authors showed that chemotherapeutic agents induced PD-L1 translocation into the nucleus, and subsequently triggered pyroptosis in breast cancer cells [113]. Thus, it becomes necessary to elucidate the effectiveness of pro-pyroptotic agents in inducing acute inflammatory immune responses in the TME.

Additional gasdermin proteins, such as GSDMA and GSDMB, also contain a pore-forming domain, and a recent study demonstrated that GZMA-driven cleavage of GSDMB was capable of inducing pyroptosis [114]. Treatment with a single round of NP-GSDMA3 and Phe-BF3 in combination was effective when an anti-PD1 antibody was concomitantly administered [115]. These data demonstrate that controlled activation of pyroptosis in tumor cells can induce anti-tumor immunity, an effect that can be enhanced by immune checkpoint blockade.

Overall, these data highlight that pyroptosis can be mediated by GSDMD and GSDME, and these proteins are important components of innate immunity, having both beneficial and adverse roles depending on the prevalent conditions. Thus, better understanding of the gasdermin family may lead to improved treatment of cancer. The lytic and immunogenic nature of pyroptosis might be an ideal attribute for cancer immunotherapy because lysis of cancer cells ensures their demise, and immunogenic elicitation of the immune system might further accelerate destruction of cancer cells.

### Therapeutic effects of NLRP3 inflammasome components

Since an aberrant activation of the NLRP3 inflammasome is implicated in cancer initiation, there is great clinical interest in the development of potential inhibitors of NLRP3 inflammasome. Recent investigations have disclosed various inhibitors of the NLRP3 inflammasome pathway which were validated through in vitro studies and in vivo experiments in animal models. Some of these inhibitors directly target the NLRP3 protein whereas others target components and products of the inflammasome [116].

In the case of breast cancer, chemotherapy and radiotherapy are therapeutic options used as first line treatment. It has been shown the ability of the immune system to contribute to the success of chemotherapy and radiotherapy.

It was demonstrated that ATP released from dying tumor cells as a result of chemotherapy can act on P2X7 purinergic receptors (P2RX7) (most potent activator of the NLRP3 inflammasome) and trigger the activation of the inflammasome. Anthracycline treated breast cancer patients carrying a loss-of-function allele of *P2RX7* developed metastatic disease rapidly compared to individuals bearing the normal allele [117]. In addition, two chemotherapeutic agents, gemcitabine and 5-fluorouracil (5-FU), activated NLRP3 inflammasome, followed by IL-1β and dendritic cells production that impaired the efficacy of chemotherapeutic drugs [118]. In line with these findings, the activation of P2RX7 combined with anti-PD-1 therapy allows tumor regression followed by immune memory response in syngeneic immunocompetent murine models [119].

Moreover, the levels of ATP are determined by CD39 [120], who plays an immunoregulatory role by modulating effector and regulatory T cells, macrophages, NKs and MDSCs, among other mechanisms [121]. In particular, Li et al., using syngeneic and humanized tumor models including HER2^+^mammary cancer models, showed that combination of CD39 and anti-PD1 blockade was dependent on the activation of the NLRP3 inflammasome in macrophages and the subsequent release of inflammatory cytokines [122]. Importantly, CD39-targeting agents have recently entered clinical trials.

Jin et al. reported that metastatic breast cancer cells were able to secrete high levels of ATP that enhances tumor invasion and tumor growth by inducing inflammasome activation in a P2Y purinergic receptor 2 (P2Y2R)-dependent manner, and radiotherapy-resistant-MDA-MB231 cells showed increased inflammasome activation in a P2Y2R-dependent manner [121]. The same authors recently determined that inflammasome components can be regulated by the P2Y2R activation and are involved in tumor progression evidencing that NLRP3 and caspase-1 mRNA levels were upregulated in radiotherapy-resistant breast cancer cells [123].

As NLRP3 activation causes pyroptotic, immunogenic cell death and the release of pro-inflammatory factors, direct inflammasome activation within the tumor may be an important mechanism to engage antitumor immunity [96].

Recently, Huang et al. have demonstrated that the lipid and protein phosphatase PTEN, a known tumor suppressor, directly interacts with NLRP3 and dephosphorylates it to enable NLRP3–ASC interaction, inflammasome assembly and activation, and myeloid PTEN can determine chemotherapy responsiveness by promoting NLRP3-dependent antitumor immunity [124].

Inhibitors of the IL-1 signaling pathway that block the IL-1α or IL-1β receptor are the most efficient examples of therapies used in breast cancer targeting the NLRP3 inflammasome [125]. Among these, anakinra, a recombinant non-glycosylated human interleukin-1 receptor antagonist (IL-1Ra), inhibits both IL-1α and IL-1β signaling. Canakinumab, a human monoclonal antibody, targeted at IL-1β, and rilonacept, a dimeric fusion protein consisting of the ligand-binding domains of the extracellular portions of the human interleukin-1 receptor component (IL-1R1) and IL-1 receptor accessory protein (IL-1RAcP) linked in-line to the fragment-crystallizable portion (Fc region) of human IgG1, are clinically approved [126].

Clinical trials to verify if anakinra is able to decrease tumor inflammation and improve outcomes in metastatic breast cancer patients are underway, so far showing promising preliminary data [127]. In fact, anakinra given during chemotherapy resulted to be safe and effective to up-regulate cytotoxic/NK cell transcriptional pathways and to down-modulate innate inflammation in these patients [128].

In a pilot clinical trial, including 11 patients with high-risk HER2-negative breast cancer, the use of daily subcutaneous anakinra for 4 months in combination with chemotherapy was not associated with adverse events. Transcripts of IL-1R1, MyD88, and IL-1βwere decreased during anakinra and chemotherapy treatment, while no altered expression and function of NKs and T cells was observed [129].

Moreover, in tumor-naive mice, a single dose of anakinra was able to reduce osteoclast and osteoblast activity, as well IL-1β expression [130]. Similarly, using a humanized model of spontaneous breast cancer metastasis to bone, Tulotta et al. demonstrated that anakinra or canakinumab reduced metastasis and the number of tumor cells shed into the circulation [131]. Additionally, trametinib (a MEK1 inhibitor) was capable of inhibiting NLRP3 inflammasome activation and reducing breast cancer metastasis to bones [129].

In an attempt to further validate the role of the NLRP3 in breast tumor growth, it was observed that blockade of IL-1β receptor promotes apoptosis and prevents cell cycle progression in cancer cells [132].

Interestingly, the overexpression of the anti-apoptotic protein BCL2 triggers activation of proinflammatory IL-1β and the NLRP3 inflammasome and consequent perturbation of mitochondrial integrity [133]. A study demonstrated that treatment of breast cancer cells with BCL2 inhibitors (venetoclax and WEHI-539) reduces mitochondrial fusion dynamics in the absence of cell death [134], and demonstrated that additional targeting of glycolysis with 2-deoxy-D-glucose can limit the progression of both ER^+^ and triple-negative breast cancer [135]. Therefore, we can suggest that venetoclax and WEHI-539 may be considered as two indirect activators of IL-1β and of the inflammasome because these two drugs, although they do not act directly on IL-1β and the NLRP3, inhibit BCL2 that, in turn, activates IL-1β and NLRP3.

Recently, it was described an important relationship between IL-1α and inflammation and cancer stem cell (CSCs) in HER2 + breast cancer [136]. The researchers demonstrated that, in breast cancer, a strong correlation was observed between IL-1α/IL-6 expression and the CSCs phenotype. This, in turn, enhanced activation of NF-κB and STAT3 signaling. Hence, pharmacological blockade of IL-1α signaling reduced the population of CSCs in the tumors and improved chemotherapeutic efficacy [137]. It was recently described that IL-1R signaling could suppress mammary tumor cell proliferation in the MMTV-PyMT breast cancer mouse model. Moreover, patients with breast cancer expressing high levels of IL-1α had better prognosis than those with lower levels [138].

NLRP3 inflammasome-dependent release of IL-1β induces immune cells, CD4^+^ T cells, and IL-22 expression and release, which has been associated to initiation and growth of many types of malignancies including breast cancer [139]. Approaches targeting some agents or signaling pathways bridging innate and adaptive immunity may expand the number of immunotherapy strategies able to restore or boost anti-tumor T cell responses. Among those, inflammasomes have emerged as players in cancer immunology and immunotherapy. Kaplanov et al. revealed that therapeutic blockade of IL-1β and inhibition of macrophage recruitment synergized and enabled full CD8^+^T cell activity in combination with the blockade of the T cell inhibitory molecule, PD-1, promoting significant therapeutic benefit in a murine model of breast cancer [140]. In detail, these authors demonstrated, in 4T1 tumor-bearing BALB/c mice, that the combination of IL-1β (AF-401-NA) and anti-PD1 (RMP1-14) antibodies was able to induce the complete inhibition of tumor [141]. Similar results were obtained by silencing IL-1β gene expression in cancer cells, which resulted in decreased infiltration by immunosuppressive cells (MDSCs, M2 macrophages) of orthotopic tumors, while the number of INFγ/GzmB producing CD8^+^ T cells was increased [142]. In this context, targeting tumor and host-derived IL-1β with anakinra improved anti-PD-1 therapy.

Strikingly, Zhu et al. reported that Au4.5 nanoparticles could function as vaccine adjuvants to increase antibody production by triggering NLRP3 inflammatory to mediate caspase-1 maturation and promote IL-1β secretion [143]. These studies highlighted the therapeutic potential of the NLRP3 inflammasome, and its capacity as immunotherapy response predictor.

Upon antibody-dependent cellular phagocytosis (ADCP), macrophages inhibit NK cell-mediated antibody-dependent cellular cytotoxicity (ADCC) and T cell cytotoxicity in breast cancer [144]. Upon activation, inflammasome upregulates PD-L1 and IDO to cause immunosuppression [145]. Combined treatment with anti-HER2 antibodies and inhibitors of PD-L1 and IDO enhances anti-tumor immunity and anti-HER2 therapeutic efficacy in vitro, as well as, in mouse models of HER2^+^ breast carcinoma. Additionally, neoadjuvant trastuzumab treatment significantly upregulates PD-L1 and IDO on TAMS from HER2^+^ breast cancer patients, correlating with poor trastuzumab responses [145].

Treatment with a pharmacological inhibitor of caspase-1 and gene silencing of *NLRP3* prevented leptin—induced growth of breast cancer cells via promotion of cell cycle progression and suppression of cell apoptosis [146]. According to these findings, the therapeutic approach with of NLRP3 and caspase-1 (MCC950 or Ac-YVAD-cmk) inhibitors is able to attenuate breast cancer cells growth [147] demonstrating that caspase 1 blockade can inhibit inflammatory responses regulated by both cytokines- and pyroptosis-dependent inflammasome.

It has been demonstrated that metabolic and caspase-1 activities in TAMs are linked. Caspase-1 inhibitors (z-WEHD-FMK and Ac-YVAD-CMK) suppressed peroxisome proliferator-activated receptors (PPAR)γ cleavage, lactate secretion and breast tumor growth. These inhibitors increase fatty acid oxidation, leading to the inhibition of intracellular aggregation of lipid droplets and decreasing the differentiation of TAMs [148]. It has recently been shown that transcription factor EB (TFEB) activated the transcription of PPARγ, which in turn blunts NFκB activation, culminating in downregulation of NLRP3/IL-1β/IL-6 axis and enhanced autophagy and lysosome activities. Specifically, analysis of breast cancer patient tumor genome database demonstrated that TFEB suppressed a wide of molecules in TAMs. Hence, it can represent a promising therapeutic target for breast cancer [149].

It is well known that breast cancer susceptibility gene 1 (*BRCA1*) is the major breast cancer suppressor gene, which encodes a protein critical for maintaining DNA integrity and genomic stability. Its deficiency impairs mitophagy that, when is defective, leads to the accumulation of damaged mitochondria and excessive ROS, which then activates the NLRP3 inflammasome [150].

Treatment with carbonyl cyanide 3-chlorophenylhydrazone (CCCP), a mitochondrial uncoupler, generates more cleaved caspase 1 and mature interleukin-1β (IL-1β) in BRCA1-knockdown cells confirming that BRCA1 deficiency can trigger inflammasome activation and establish a tumor-associated microenvironment that favors cancer progression. In fact, the treatment with glibenclamide, an inflammasome inhibitor, delayed tumor recurrence, blocked lung metastasis of the *Brca1* mutant tumors, reduced the percentage of macrophages in relapsed tumors, and the mRNA levels of M2 macrophage markers (CD163, CD206, and Mgl2) in recurrent tumors [151].

In breast cancer, particularly in TNBC, TILs are positively correlated with a favorable prognosis, while the presence of CD8^+^ TILs, including a subpopulation of tissue-resident memory T (T_RM_) cells, is associated with increased response rates to anti-PD-1 antibodies [152]. Moreover, NLRP3 exhibits significant association with the expression of immune checkpoint genes, suggesting that the inflammasome may be associated with response to immunotherapy [153]. In this context, a better understanding of the immune contexture and of its modulation by malignant cells can lead to therapeutic combinations in breast cancer, especially in TNBC.

Another important point of discussion concerns the crosstalk between NLRP3 inflammasomes and the administration of immune checkpoint inhibitors (ICIs), such as programmed death-1 (PD-1)/programmed death-ligand 1 (PD-L1) and cytotoxic T-lymphocyte antigen 4 (CTLA-4) [154]. In the context of cancer therapies, inflammasomes play an anti-tumoral role in response to immunogenic chemotherapy [155] and to BRAF inhibitor in melanoma [156]. Furthermore, a mutational analysis performed on a subset of patients that developed hyper progressive disease (HPD) after pembrolizumab identified the presence of missense or indel mutations in genes involved in the negative regulation of NLRP3 activation and inflammasome pathway [155].

Inflammasome activation and the induction of pyroptosis upstream of IL-1β release could render tumors more sensitive to therapy [154]. However, only 15–60% of patients respond to immune checkpoint blockade due to, among other things, a lack of CD8^+^T-cells infiltrating the tumor microenvironment. Researchers are exploring combination therapies that facilitate CD8^+^T-cell recruitment in order to sensitize tumors to immune checkpoint blockade [156, 157]. It is known that immune cell infiltration is regulated by tumor-intrinsic oncogenic and epigenetic pathways. In this context, it is crucial to identify small molecules that could convert immunologically “cold” tumors into “hot” tumors [158], particularly in aggressive cancers such as TNBC, and thereby modulate PD-1/PD-L1 expression. In line with this strategy, an investigational phase I clinical trial in patients with solid cancers has recently been initiated with the NLRP3 agonist BMS-986299 to study its therapeutic potential as a pyroptosis-stimulating compound alone or when administered in combination with the checkpoint inhibitors nivolumab and ipilimumab (ClinicalTrials.gov Identifier: NCT03444753).

Based on these promising results, the characterization of molecular targets to trigger inflammasome activation is needed to design selective and potent inhibitors.

## Conclusions

Given the complex NLRP3 signaling cascade, a variety of agents can be considered for its inhibition. Several types of inflammasome inhibitors, as well as their components are being developed. The utilization of therapeutic strategies against the NLRP3 inflammasome to block breast cancer progression depends on the different molecular subtypes and patient heterogeneity. The decision to combine IL-1β inhibition and other drugs needs a more detailed understanding of the tumor immune microenvironment including the immune cell subtypes, as well as role of IL-1 on these cells. In addition, with the complexity and inter and intra-tumor heterogeneity of breast cancer, some unanswered question is whether the role of inflammasome in TNBC is similar or different compared to other molecular sub-types. For this reason, it is necessary to focus on TNBC patients and evaluate if they have best responses after treatment with NLRP3 inflammasome inhibitors. On the other hand, pyroptosis and the compounds that inhibit this mechanism have been intensely implicated in cancer. It is assumed that pyroptosis results in different immunological outcomes, mediating several inflammatory responses that will depend on multiple mechanisms, including the type of activated gasdermin, as well as the mechanism of cell activation. Inflammasome signaling pathways are diverse among tumor types, and the understanding on how to manage this diversity is extremely important for translational research, with the possibility of new therapeutic targeting of tumorigenesis.

## Data Availability

Not applicable.

## Abbreviations

AIM-2: Absent in melanoma 2
ATP: Adenosine-triphosphate
CAFs: Cancer associated fibroblasts
CLRs: C-type lectin receptors
CSCs: Cancer stem cells
CTLA-4: Cytotoxic T-lymphocyte antigen 4
DAMPs: Damage-associated molecular patterns
DCs: Dendritic cells
ER: Endoplasmic reticulum
ER: Estrogen receptor
FADD: (FAS)-associated death domain
FAS: Fatty acid synthase
GSDMD: Gasdermin D
HAMPs: Homeostasis-altering molecular processes
HER2: Human epidermal growth factor
ICD: Immunogenic cell death
IL-1β: Interleukin-1β
IL-18: Interleukin-18
LC3B: (Microtubule-associated protein 1 light chain 3B)-positive puncta
LPS: Liposaccharide
MAMPs: Microbe-associated molecular patterns
MDSCs: Myeloid-derived suppressor cells
MSCs: Mesenchymal stem cells
MyD88: Myeloid differentiation primary response 88
NF-kB: Nuclear factor-kB
NKs: Natural killer
NLRs: Leucine-rich repeat–containing receptors
NLRP3: NOD-, LRR- and pyrin domain-containing protein 3
NODs: Nucleotide-binding oligomerization domain
PD-1: Programmed death receptor 1
PRRs: Pattern recognition receptors
PR: Progesterone receptor
PTMs: Post translational modifications
PYD: Pyrin domain
RIP1: Receptor-interacting protein 1
RLRs: RIG-I-like receptors
ROS: Reactive oxygen species
TAMs: Tumor associated macrophages
TAK1: Transforming growth factor 1 activating kinase
TCGA: The Cancer Genome Atlas
TILs: Tumor-infiltrating lymphocytes
TLRs: Toll-like receptors
TME: Tumor microenvironment (TME)
TNBC: Triple-negative breast cancer
TNF: Tumor necrosis factor
TRM: Tissue-resident memory T
